# “They Are Worth Their Weight in Gold”: Families and Clinicians’ Perspectives on the Role of First Nations Health Workers in Paediatric Burn Care in Australia

**DOI:** 10.3390/ijerph18052297

**Published:** 2021-02-26

**Authors:** Julieann Coombes, Sarah Fraser, Kate Hunter, Rebecca Ivers, Andrew Holland, Julian Grant, Tamara Mackean

**Affiliations:** 1 The George Institute for Global Health, University of New South Wales, 2052 Sydney, Australia; khunter@georgeinstitute.org.au; 2 School of Population Health, University of New South Wales, 2052 Sydney, Australia; sarah.fraser@unsw.edu.au (S.F.); rebecca.ivers@unsw.edu.au (R.I.); 3 The University of Sydney, 2052 Sydney, Australia; andrew.holland@health.nsw.gov.au; 4 Charles Sturt University, 2795 Bathurst, Australia; jugrant@csu.edu.au; 5 Flinders University, 5000 Adelaide, Australia; tamara.mackean@flinders.edu.au

**Keywords:** First Nations, health workers, burn aftercare, children, Australia

## Abstract

Burns affect Australia’s First Nations children more than other Australian children, they also experience longer lengths of stay in tertiary burns units and face barriers in accessing burn aftercare treatment. Data sets from two studies were combined whereby 19 families, 11 First Nations Health Worker (FNHW) and 56 multidisciplinary burn team members from across Australia described the actual or perceived role of FNHW in multidisciplinary burn care. Data highlighted similarities between the actual role of FNHW as described by families and as described by FNHW such as enabling cultural safety and advocacy. In contrast, a disconnect between the actual experience of First Nations families and health workers and that as perceived by multidisciplinary burn team members was evident. More work is needed to understand the impact of this disconnect and how to address it.

## 1. Introduction

Australia is made up of distinct groups of Aboriginal and Torres Strait Islander peoples, each group has their own culture, language, beliefs and practices. Aboriginal and Torres Strait Islander peoples are the first peoples of Australia, and there is evidence of their existence in Australia many thousands of years prior to European colonisation. They are acknowledged and respected as the Traditional Custodians of the Land.

The authors of this manuscript recognise the two distinctive First Nations populations of Australia; Aboriginal and Torres Strait Islander people as being the First People of Australia. This study has taken place across Australia on First Nations land, the term First Nations people has been used when referring to Aboriginal and Torres Strait Islander people of Australia in this manuscript. 

First Nations health workers are employed in tertiary health settings around Australia to improve cultural safety and enhance interactions between First Nations people and the Australian healthcare system [1]. Studies demonstrate improved health outcomes and communication when FNHWs are involved in care in tertiary healthcare settings [2,3]. Other studies also show that involvement of FNHWs in care has led to improved access to aftercare for children with chronic conditions [4,5]. 

Burns can be a devastating injury causing long term physical and emotional trauma [6,7]. Australia’s First Nations children are admitted to hospitals as a result of a burn injury twice as often as other Australian children and have longer lengths of stay [8]. Serious burn injuries need specialised acute tertiary care and often require multidisciplinary aftercare for extended periods of time [6]. Given the reported benefits of their involvement in other care contexts [9], it is clear that FNHWs are important facilitators to the continuity of burn aftercare once a First Nations child has left a tertiary healthcare setting. 

Burn care is informed by Western biomedical concepts of health [10], despite First Nations family needs for best quality healthcare. Racism towards First Nations people in Australia has been a reality since colonisation and brings with it a power imbalance that is entrenched in our health systems and leads to poor health outcomes. 

Quality healthcare is such that it incorporates First Nations concepts of health and healing and delivery of care by FNHWs [11]. However, how FNHWs work in the healthcare system at the interface of Western biomedical care and First Nations concepts of health and healing in relation to childhood burns is unclear. 

To our knowledge, this is the first study to describe the role and function of FNHWs in multidisciplinary burn care for Australia’s First Nations children. The desired, actual and perceived role of FNHWs is described by families and the FNHW themselves, along with data from the perspectives of multidisciplinary burn team members.

## 2. Methodology and Methods 

### 2.1. The Coolamon Study

This work comprises a substudy of the Coolamon Study, which examined a range of factors related to care and impact of burns in Aboriginal and Torres Strait Islander children who sustained a serious burn. As part of this study, Australia’s First Nations children under the age of 16 years who had sustained a burn injury and present to a tertiary pediatrics burn unit were recruited from New South Wales, Queensland, South Australia and Northern Territory. Data collected from this study included sociodemographic information, out of pocket costs, functional outcome and measures of pain, itch and scarring. The study also explored barriers and facilitators of burn after care for the family. Health-related quality of life was measured using the PedsQL, and impact of injury using the family impact scale. Clinical data and treatment were also be recorded [12]. Within this larger study two sub-studies were conducted by JC and SF undertaking their PhDs [12]. 

The Coolamon Study [12] comprises four sub-studies. This paper reports on data from two sub-studies led by authors JC and SF to better understand the role and contribution to care by FNHWs in multidisciplinary burn care for First Nations children, data from the two sub-studies were combined. Data included incorporates the perspective of families on the perceived and desired contribution to burn aftercare by FNHWs from sub-study one. Data from stub-study two includes the descriptions of FNHWs’ involvement in multidisciplinary burn care from the perspectives of the workers themselves and of multidisciplinary burns team. The combining of the two data sets is essential in facilitating a comprehensive understanding of the role of FNHWs from multiple perspectives and lived experiences regarding burn care for First Nations children and their families. Approval was granted by ethics committees in each state research was undertaken, as well as all the relevant Aboriginal health ethics departments. These include the Aboriginal Health Research Ethics Committee (South Australia; EC00185), Aboriginal Health & Medical Research Council Ethics Committee (New South Wales; EC00342), Human Research Ethics Committee for the Northern Territory Department of Health and Menzies School of Health Research (EC00153), Central Australian Human Research Ethics Committee (Northern Territory; EC00155), Women’s and Children’s Health Network Human Research Ethics Committee (South Australia; EC00197), Sydney Children’s Hospitals Network Human Research Ethics Committee (New South Wales; EC00130), The University of Queensland Medical Research Ethics Committee (EC00179), Children’s Health Queensland Hospital and Health Service Human Research Ethics Committee (EC00175), Townsville Hospital and Health Service Human Research Ethics Committee (Queensland; EC00183). 

### 2.2. Analysis Approach

#### 2.2.1. First Sub-Study

The first set of data was collected by the first author J.C., an Australian First Nations researcher, using indigenous research methodologies. The first author, J.C.’s worldview as a Murri woman impacted her standpoint in this sub-study [13,14]. J.C. sought to understand the barriers and/or facilitators in accessing burn aftercare using indigenous methods of yarning [15] and Dadirri [16]. A total of 59 individuals from 18 different families were asked to share their burn care journeys. Families were recruited purposively from the larger national study examining burn care in Australia’s First Nations Children, the Coolamon Study [12]. Families were selected to ensure diversity of experience and access to burn aftercare. Families resided in communities across Australia and the Torres Strait Islands. These stories were audio recorded and transcribed verbatim. Families received a copy of their transcript and a follow up phone call was made to ensure the family’s stories was accurately reflected. Data were analysed using a cyclical process which gave ownership of the story to the storyteller and empowered the voice of each family [14]. 

#### 2.2.2. Second Sub-Study

Interface research methodology [17] incorporating both Indigenous and Western biomedical knowledges guided the second sub-study for author SF, a non-Indigenous researcher. Sub-study two sought to explore how burns care is delivered, with a focus on the care of First Nations children and families. It also investigated factors informing burn care and explored how clinicians in burn teams use guidance documents and if such documents are appropriate for care of First Nations children. In the second sub-study, and using a semi-structure interview guide, author SF interviewed 76 healthcare professionals from six different multidisciplinary burn teams across five jurisdictions in Australia. Of the 76 participants interviewed, 11 were employed in specific First Nations health worker roles. These included one Aboriginal health practitioner and 10 Aboriginal or Indigenous liaison officers. Interviews were also audio recorded, transcribed verbatim and participants confirmed the transcripts were accurate and true. Data that discussed the specific role, either perceived or actual, of FNHWs have been included here. All other data was excluded for this analysis.

Individually authors J.C. and S.F. thematically analysed their own data and then came together to consolidate their findings and discussed over all themes with the third author TM who has expertise in Indigenous methods and from there reached consensus on the key findings and research themes.., The coming together by the two researchers and their respective methodologies, provides an opportunity to demonstrate how research at the interface might deliver outcomes that marry Indigenous ways of doing and Western biomedical care. This is not unlike Ganma, described by the Yolgnu people as respectful two-way sharing of cultural knowledge and interaction between Aboriginal and non-Aboriginal people [14]. J.C., S.F. and T.M. met to discuss the data and existing themes, grouped the data (as per result below) and then synthesised all information specifically into three categories: (i) First Nations children’s and families’ perspectives (ii) First Nations health workers’ perspectives and (iii) multidisciplinary burn team member’s perspectives.

## 3. Results

Results from both sub-studies highlighted the role of FNHWs in delivery of burn care, from a patient and family perspective (study 1) and from a clinician perspective (study 2). The data have been arranged into three sections—the FNHWs’ perspectives of care, the multidisciplinary burns team perspectives of the role, and the First Nations families’ perspectives of the FNHWs’ role. Quotes have been used throughout to illustrate the themes that were synthesised from the data and pseudonyms used to protect identities where necessary.

### 3.1. First Nations Children’s and Families’ Perspectives

Remote participants needing multidisciplinary teams for ongoing burn aftercare described the importance of the FNHWs in supporting children and families accessing burn aftercare. Families recounted how FNHWs provided access to essential tangible support and a culturally safe environment. Families also identified challenges related to the role of FNHWs and their availability to be involved in their child’s care (Table 1). Several sub-themes were identified within these aspects of care.

#### 3.1.1. Tangible Support

FNHWs were able to assist families with tangible support in transporting the child and family to appointments, with short- and long-term accommodation, food and taxi vouchers and filling in forms for the patient assistance transport scheme.

*“He needs to still see the physio. No, I don’t have a car, we either catch the bus to the hospital or the health girls [FNHW] came and picked us up.”*

*“She [FNHW] took off my PATS [Patient Assistance Transport Scheme] form and faxed it off to me”.*

*“The [First Nations] health workers, they brought me up a few times for our appointments, I think a couple of times.”*

*“We didn’t have anywhere to stay but she helped us with a place while he had his dressing done… every few days we’d go back to the hospital with taxi vouchers she gave us.”*

#### 3.1.2. Cultural Support

Families voiced how dislocation from home and community was understood and valued by the FNHW. Due to FNHWs’ advocacy between the multidisciplinary team, health services and the family, a connection was created between First Nations families and FNHWs.

*“I ended up giving her a gift after she [FNHW] done what she did, she supported me a lot and kept an eye on him and, so, I done her a nice painting she took home. She loved it.”*

*“Linda liked her [FNHW] she was happy when she saw her the next time we went for dressing ‘cause we trusted her.”*

*“She [FNHW] understood where we come from and how different it is down here they [multidisciplinary team] don’t understand.”*

*“We had to move from our community and no family is here but yeah, she [FNHW] helped me with like food vouchers, and sit down and have a cup of coffee and a yarn at the hospital house they sent us to so Maison could have dressings.”*

#### 3.1.3. Challenges and Fears

Data showed that some children and families were frustrated that FNHWs were not available when they needed them. This was often because they were either busy with other patients or there wasn’t a FNHW employed at the service. When FNHWs were available to provide support, family challenges and fears were alleviated. Although some families did not receive support from a FNHW it was express that they would have liked to have the support.

*“No, but I wish I did see one [FNHW] then she could have been with me when mum couldn’t.”*

(child)

*“I don’t think I seen anyone else—any Aboriginal worker they said they had one but was sick.”*

(mother)

*“Yes I did see an Aboriginal worker… Once for about 20 min. She was going to come back but she didn’t come back. Obviously it was flat out.”*

(mother)

*“It was so good having her [FNHW] there when they were talking them big words she would tell us what they meant.”*

(mother)

*“The Aboriginal [liaison] worker helped get my family together down here… going to be here for months in Ronald Macdonald house… we were all living in separate places… so hard on Damien’s father.”*

(mother)

### 3.2. FNHWs Perspective on Their Contribution to Burn Care

FNHWs were essential to the cultural safety of the child and the family, often acting as advocates between family and medical staff. There was a clear message from FNHWs that the need for cultural awareness training for non-Indigenous health workers was imperative in providing cultural safety and support for First Nations families. Data showed the provision of two main areas of support by FNHWs to include tangible and cultural support. Data also highlighted the importance of working together and the challenges associated with the role (Table 2).

#### 3.2.1. Tangible Support

Transport for the child and family was difficult from remote communities, and FNHWs would organise the crucial transport for the child to receive ongoing burn aftercare, alleviating some of the stress family were often face with.

*“But if it’s an outpatient we’ll sort that out as well and help the family. We used to help them with taxi vouchers but now taxi voucher are getting really, really scarce and it’s really frustrated because we get families who missed their flight, and I’m not going to tell a family catch a bus and go from here to there, it’s not fair. So we have to fight for taxi vouchers.”*

(Aboriginal Liaison Officer)

*“So at the moment my role is, I give health education to patients, I help them understand why they’re in the hospital, I help with any issues around social stuff within reason because the ILOs [Indigenous Liaison Officers] are employed for that role but because sometimes they’re short staffed… our roles overlap a little bit and that’s the whole role of the AHP [Aboriginal Health Practitioner] is the primary health care, is the holistic approach to health so sorting out everything. I also advocate for patients who need spiritual healing or cleansing, unfortunately (this hospital) don’t provide that service or support that service so I try my best to get that patient to communicate and navigate through the system with having that need addressed, the spiritual need addressed as well the Western medical need.”*

(Aboriginal Health Practitioner)

*“…Aboriginal Liaison Officers who are based on the ground floor and they do outpatients but it’s more escorting them to appointments, booking them back home and stuff… those guys [patient/family] if it was a burns clinic outpatient appointment they probably wouldn’t receive really any support without this.”*

(Aboriginal Liaison Officer)

#### 3.2.2. Cultural Support

Supporting First Nations families through cultural connection helped with effective communication between multidisciplinary team and families. This was expressed by FNHWs as an important and essential component of their role.

*“Cultural advice you know or cultural safety on the clients say if they, we do a research and find out the language, where they’re from and what traditions they have in their community and if they, you know some of the patients don’t want to be seen by females so we have to let them know. Just making sure that staff are safe too and the patient’s safe.”*

(Aboriginal Health Practitioner)

*“… where the patient really fully understands or the patient’s parent or guardian fully understands what’s going on with a particular patient and will make an informed decision around that patient’s care. So there’s a lot of cultural barriers around that and you know like for me I think our cultural awareness training really lets staff down because it only addresses the first part of the cultural continuum of going towards patient cultural safe, delivering a culturally safe service...”*

(Aboriginal Health Practitioner)

*“English is probably their third language it’s still funny though, I don’t know how to explain it but we can still connect.”*

(Aboriginal Liaison Officer)

#### 3.2.3. Two Worlds Working Together

FNHWs functioned as advocates for the child and family and as mediators when working with the multidisciplinary burns team. There was recognition that FNHWs and the multidisciplinary team need to work together for the benefit of the child and family in order to contribute to better healing outcomes.

*“I think if you understand what the organisation expects and you understand the upbringing that you were raised in and you balance them out you realise how you can actually go about it and bring both of the worlds together and that means the outcome for the families is, you’re going to achieve something.”*

(Aboriginal Liaison Officer)

*“ I’ve just always been asked can you come and talk to them, or they’re not going to turn up for their physio appointments or they’re not getting out of bed for me can you go, them kind of things but I’ve never really been asked culture-wise stuff whether it’s because they’ve been around long enough they’ve got that knowledge and experience, but yeah I’ve just sort of being asked, they’re not getting up, they’re swearing, behaviour, the parents need to turn up, that sort of stuff.”*

(Aboriginal Liaison Officer)

*“Just letting the team know like if we get someone from remote we let the team know where this community is, how far the nearest hospital is because there’s only usually clinics on the communities and so looking at things geographically and then like what their cultural background is because all Aboriginal cultures are different but same if you know what I mean. Yeah and just basically looking after them while they’re here and making their stay comfortable and their journey here comfortable.”*

(Aboriginal Liaison Officer)

*“We go there and just advocate on behalf of the Aboriginal patient and have feedback, it’s in regards to the feedback, more of the patient.”*

(Aboriginal Liaison Officer)

*“So when a family comes up for outpatients if they need a bit of extra support, they’ll contact the social worker or myself.”*

(Aboriginal Liaison Officer)

#### 3.2.4. Challenges at Work

The lack of FNHWs employed was stated as an important contributing factor in not being able to deliver best practice burn care. FNHWs expressed their concerns that staff fatigue and subsequent assumptions resulted in multidisciplinary workers treating First Nations patients unjustly.

*“So fatigue management also comes into it because then the staff get tired and a patient comes in as a new admission so that new admission is not treated as a new admission because it’s easier to go, actually this patient reminds me of the last patient that came here and they weren’t very engaged so straight away, and it happens, that’s a natural response when someone’s fatigued.”*

(Aboriginal Health Practitioner)

*“… we need more Aboriginal people in here especially AHPs [Aboriginal Health Practitioners]. I can just see a whole system of AHPs working across the hospital, less money because we’d be doing things right from the start, not putting down any other profession it’s not about that it’s about delivering a service. You know when we look at customer service if we look at big corporations that make lots of money, what do they do, they really focus on what their target group want and need. That’s what it’s all about.”*

(Aboriginal Health Practitioner)

*“I’m one to a whole division so no-one thinks to refer sometimes, maybe they’re afraid to refer because they might be seen as not being able or culturally competent to deal with that patient. And so there’s one of me to the division so that’s very tricky most of the time.”*

(Aboriginal Health Practitioner)

### 3.3. Multidisciplinary Burn Team Members’ Perspectives

Multidisciplinary burn team members have a varied understanding of the importance of the FNHW role. Perceived descriptions of the care provided by the FNHW role including meeting tangible and cultural support needs (Table 3).

#### 3.3.1. Tangible Support

Multidisciplinary team members understood the role of FNHWs was to provide families with tangible support including provision of education and networking for aftercare appointments. They also perceive the role is to stop people from absconding against medical advice.

*“We don’t have an Aboriginal Liaison Officer [ALO] at the moment because she’s unwell but we are recruiting to a contract position and I would always take the ALO… with me to go on the first visit with a family, check the family, because I just think it’s really useful and then we kind of divide the tasks so you know she might do some of the more liaison practical things and I will do the trauma stuff with the family and the assessment.”*

(Social Worker)

*“I use them [Aboriginal Liaison Officer] all the time especially if patients abscond or I need to know more about family relationships like, do you know much about this mob or their family… And I also use them quite a bit when you’re trying to arrange and negotiate appointments for outpatient clinics and where to from here.”*

(Clinical Nurse Consultant)

*“… it is such a isolating environment down here, very different and I don’t pretend to know how I can understand and address those things culturally and so I really look for their [FNHW] input so to try and get an understanding how the parents and the child’s feeling and what their care needs are.”*

(Medical Consultant, Surgeon)

*“We have one or two chronic rehabilitation patients post-burn who are Indigenous, they are very involved with the ALOs [Aboriginal Liaison Officers] and also very involved with social work in terms of helping to arrange transport particularly if they’re from regional centres, to us and back from us.”*

(Registrar)

#### 3.3.2. Cultural Support

Multidisciplinary team members perceive the role of the FNHW to include the provision of cultural support including an appreciation of family circumstance and communication. Some team members stated the importance of having FNHW involvement to ensure the family and child from remote communities felt comfortable.

*“I’ve been in this hospital in different capacities over time from a very junior doctor to a registrar and now a consultant and I have noticed that there’s been a difference in the input from our Aboriginal Liaison Officers and I’d like to see more input because when I was a junior doctor I remember them being there all the time on the ward with the families and you could really notice the comfort that families found from having cultural similarities with staff there.”*

(Consultant)

*“We always make sure we get the Indigenous liaisons involved just simply because a lot of the time especially if they’re coming from remote communities they may not have the family support and we know that family and community is a big thing for Indigenous people.”*

(Occupational Therapist)

#### 3.3.3. Two Worlds Grappling

Multidisciplinary burn team members understood the importance of working together and the need for FNHW involvement in burn care. However, there was evidence in the data that illustrated a lack of commitment towards involving FNHWs in burn team activities such as core multidisciplinary meetings. At the same time often criticising that FNHWs are unable to effectively relay important family information.

*“… I think both work, you can’t have one [multidisciplinary team] without the other [FNHW] and I think probably there are ILOs [Indigenous Liaison Officers] being underutilised and under-resourced for the amount of demand that we have and certainly like the model of care in Queensland for our ILOs are more a supportive role, they don’t take on a clinical caseload, they don’t do hands on dressing changes or anything like that, they’re more an emotional support and navigating the health system for the Aboriginal clients and Torres Strait Islander clients.”*

(Occupational Therapist)

*“I think the ideal thing would be to get Aboriginal staff involved in their management, they sort of identify better with Aboriginal staff, the difficulty is finding those who are trained well enough but they do seem to respond better to those than us telling them something.”*

(Consultant)

*“… sometimes we have to get the Aboriginal Liaison person in if we’re having difficulty communicating, especially the kids that come down… sometimes it’s really hard to engage with them, and you’ve got to get help in for that sort of thing. The indigenous kids we tend to get here coming from… tend to be different.”*

(Surgeon)

*“I think their ability to communicate with the team has been lacking, maybe they’re great at talking to the family but then feeding back and actually feeding our information back is probably, there’s not a really strong link there. So from my perspective I think having stronger ALO [Aboriginal Liaison Officer] support, that liaison and educating me of what I need to do or how I can get through the other way, how can I get my message through via the ALO officer, probably involving them more would help in certain circumstances.”*

(Physiotherapist)

*“I guess one of the obvious ones is asking for support from our ILO [Indigenous Liaison Officer] to facilitate meetings or education or discharge planning or resilience sort of stuff, to be having that supported conversation with me so that I’m being culturally appropriate and culturally aware.”*

(Clinical Nurse Consultant)

*“We have Aboriginal Liaison Officers [ALOs] in the hospital and we’re very quick to try and get them involved very early on but I think their capacity at times from my perspective has been, I think their ability to communicate with the team has been lacking, maybe they’re great at talking to the family but then feeding back and actually feeding our information back is probably, there’s not a really strong link there. So from my perspective I think having stronger ALO support, that liaison and educating me of what I need to do or how I can get through the other way, how can I get my message through via the ALO officer, probably involving them more would help in certain circumstances.”*

(Physiotherapist)

#### 3.3.4. Challenges at Work

Multidisciplinary team members recognised there are challenges between FNHW availability and being over worked due to inadequate resourcing of First Nations workers. It was also recognised that poor interaction with FNHWs by the multidisciplinary team members impacted outcomes for First Nations children.

*“When they’re identified as Aboriginal or Torres Strait Islander we would always engage with the Aboriginal Health Officer… she’s currently off at the moment and we don’t have that service available to us.”*

(Clinical Nurse Consultant)

*“… wonderful, she’s worth her weight in gold, but we need ten of her.”*

(Physiotherapist)

*“… occasionally we’ve got to get the Aboriginal and Torres Strait Islander Liaison Officer involved if it’s a particularly sticky situation but most times we can deal with things in the usual manner.”*

(Surgeon)

*“I understand it’s about getting them home and back into their environment and the financial strain and things like that about getting back but I feel we’re very mindful of those sorts of things but what we’re pushing is long term scar, long term loss of movement of arms and hands, you know for that sort of outcome, but I do think probably the relationship between her [Aboriginal Liaison Officer] and us [burns team] probably isn’t ideal which maybe then impacts onto those children.”*

(Surgeon)

## 4. Discussion

Both studies specifically explored the role of FNHWs in delivery of burn care, both acute and in aftercare. Combining the data of First Nations author J.C.’s study informed by Indigenous methodology [18] with the data from non-Indigenous researcher SF who engaged interface research methodology [17] deliberate and purposeful. This gives a breadth of context (understanding the entire patient journey) and depth of understanding (how the role participates in the team and how the roles meets the needs of the families across the journey). The combination of data provides an opportunity to further explore the role of FNHWs in burn care for First Nations children First Nations ways of knowing, being and doing in the context of health and healing are not always present within the Western biomedical health systems that surrounds and informs initial burn care and burn aftercare. Multidisciplinary teams do recognise the importance of FNHWs, but systems are not set up to support resourcing because all categories said they’re overworked. This further supports evidence about how the biomedical model excludes the lived experiences and knowledge of First Nations people [10]. This lack of inclusion is partly the result of a discrepancy between the perspectives of First Nations families, FNHW, and multidisciplinary burn team members regarding different ways of health and healing. The First Nations families experienced improved cultural safety and were able to access necessary tangible support through care provided by the FNHW. This supports other studies [9] that have highlighted the necessary role of FNHWs in better being able to understand the needs of First Nations families. Similarly, FNHW experience of being involved in burn care for First Nations families was that they understand their contribution to care and wanted to be involved in the multidisciplinary care yet were excluded. While First Nations families and health workers experienced and understood these needs, the health systems informing multidisciplinary burn care excluded First Nations ways of knowing being and doing. This was evidenced by the team’s exclusion of FNHW from team meetings, and by the Western biomedical models lacks acknowledgement of the importance of the FNHW role and subsequent resourcing. 

Different ways of knowing, being and doing exist. Values are often placed on needs without considering these differences. This is evidence of colonisation through a disregard to different ways of knowing, being and doing. Care that is regardful of differences, improves dialogue and reduces power imbalances will lead to an experience of improved culturally safe care [19]. 

### 4.1. Two Worlds Collide 

There was an expectation verbalised by FNHWs that some health professionals only used the FNHWs role as trouble shooters for assumed difficult patients and did not value the professional role of a FNHWs. However, the diverse nature of services for First Nations children and families was expressed as essential by family members. FNHWs provided cultural and spiritual care, tangible support such as transport to aftercare appointments, accommodation and food vouchers. Families valued the FNHW for alleviating fears, providing cultural safety and advocacy due to a shared recognition of cultural knowledge and connection [20].

Members from the multidisciplinary team merely asked for assistance from FNHWs when there was a ‘problem to fix’ such as absconding. Ongoing aftercare appointments were facilitated by FNHWs only when all else failed due to team members inability to reach the family for aftercare appointments. From the data collected from members from the multidisciplinary team it was apparent that FNHWs are not valued for the quality of care that families had expressed [21].

### 4.2. Colonisation

A power imbalance exists in favour of the Western biomedical model [19], especially as it relates to burn care for First Nations children in Australia [22]. Multidisciplinary burn team members showed power imbalances through their placement of value on the needs of First Nations families. Their values were assumptions, not grounded in evidence, and showcased the implicit bias within the system of multidisciplinary burn care. Furthermore, multidisciplinary burn team members sought input from FNHWs sometimes based on needs that were centred in a deficit mindset. For example, when they thought a First Nations family would not attend follow-up care due to dysfunction. The problematisation of First Nations people’s needs is indicative of structural racism in Australia’s health system [23]. 

### 4.3. Cultural Safety

Cultural safety has been shown to improve the health outcomes of First Nations people in accessing mainstream healthcare [24,25]. Furthermore, engagement in critical reflexivity in healthcare has been shown to support the competence of non-Indigenous healthcare professionals. Support for cultural competence in policy documents contributes further to improved cultural safety. Good cultural competence leads to an experience of culturally safe care. First Nations families and health workers understood the FNHW role in enhancing an experience of cultural safety, whilst multidisciplinary burn team members grappled to acknowledge the role’s contribution to achieving quality care. So, while multidisciplinary burn team members said they understood they role, the disjuncture between the two ways of knowing being and doing supports the idea that enhanced cultural training is imperative for multidisciplinary team members to move beyond the divide and contribute constructively to closing to gap.

### 4.4. Strengths and Limitations

Combining the two sets of data strengthens the reliability and validity of the issues surrounding FNHWs’ crucial involvement in burn aftercare. Furthermore, this combining of data also resonates with the methodology used to inform sub-study two whereby Aboriginal ways of knowing, being and doing [26] are integrated with the Western biomedical standpoint [19]. It has also shown that by using Indigenous methods, which is instrumental in decolonising research approach that supports the empowerment of all Aboriginal and Torres Strait Islander peoples and communities in this study [13,27,28]. A limitation of sub-study two was not asking if any multidisciplinary team member identified as a First Nations person. Sub-study one only yarned with children and their family that were admitted to a tertiary burns unit and may have missed perspectives from families whose child with a burn was seen outside of the tertiary setting.

## 5. Conclusions

Delivery of culturally safe care is essential to ensure equitable health outcomes. This paper has highlighted the importance of inclusion of FNHWs in delivery of healthcare to Australia’s First Nations children who need ongoing complex care. It is essential that FNHWs are active participants in the multidisciplinary care burn meetings and are encouraged and supported by the team members to engage in partnership of service delivery and ongoing aftercare for the child and family. This will require additional resourcing and additionally important changes to clinical hierarchies in the delivery of care, to ensure the important role of FNHWs is appropriately noted and rewarded. 

## Figures and Tables

**Table 1:** First Nations children’s and families’ perspectives.

Tangible Support	Cultural Support	Challenges and Fears
Communication/interpreters Accommodation Food Transport Help with aftercare appointments	Cultural and spiritual care Connection to people and place Cultural safety/Advocacy Trust	Lack of cultural understanding from non-Indigenous health professionals Not understanding the medical jargon when FNHW was not available Not having a FNHW at the hospital Overworked and not available Fear of child removal

**Table 2:** First Nations health workers’ perspectives on their contribution to and challenges with burn care.

Tangible Support	Cultural Support	Two Worlds Working Together	Challenges at Work
Connection to people and place Accommodation Food Transport	Cultural and spiritual care Communication/interpreters Cultural safety/advocacy Training/education	Called upon if problems with families Communication Advocacy Training/education	Not able to be involved from the start Lack of communication Working above and beyond Overworked

**Table 3:** Multidisciplinary burn team member’s perspectives and challenges.

Tangible Support	Cultural Support	Two Worlds Grappling	Challenges/Work
Fix problems such as absconding and surveillance Not understanding Education Network for aftercare appointments	Not appreciating needs of family and patient circumstances Communication	Understanding the importance of working together Perceived need for involvement Non-involvement in core Multidisciplinary team meetings	Not enough FNHWs Overworked

## Data Availability

All data collected are stored in secure servers at The George Institute and to maintain Indigenous data sovereignty cannot be placed on an Open Data Platform.
